# Note on brachypterous Stenochiini from China (Coleoptera, Tenebrionidae) with description of a new species

**DOI:** 10.3897/zookeys.415.6349

**Published:** 2014-06-12

**Authors:** Cai-Xia Yuan, Guo-Dong Ren

**Affiliations:** 1 College of Life Sciences, Hebei University, Baoding 071002, China; 2 College of Life Sciences, Yan’an University, Yan’an 716000, China

**Keywords:** Tenebrionidae, Stenochiini, *Strongylium*, new species, China

## Abstract

A checklist of 29 brachypterous species in the tenebrionid tribe Stenochiini is given for China and neighboring countries. A new species is described and illustrated under the name of *Strongylium liangi* **sp. n.** (CHINA: Yunnan). Also, some new distribution data is provided for *S. claudum* (Gebien, 1914), and a distribution map of all *Strongylium* species in the checklist is presented.

## Introduction

The East Asian brachypterous species of the tenebrionid tribe Stenochiini, including 14 species/subspecies in four genera, were revised by Masumoto (1999). Later, more species and genera were added or transferred to this group by Ando (2003), Masumoto (2006), Yuan and Ren (2006), Masumoto et al. (2007), Löbl et al. (2008), Ando and Nakahama (2009), and Masumoto et al. (2013). This group currently includes six genera and 28 species/subspecies, of which 13 species/subspecies in four genera are known to occur in China. In the present study, a new brachypterous species of *Strongylium* from Yunnan, China is described, *Strongylium liangi* sp. n. The checklist of the brachypterous species of the tribe Stenochiini from China and neighboring countries is updated and a distribution map of the *Strongylium* species is provided, including new distribution data for *Strongylium claudum* (Gebien, 1914).

## Material and methods

**Figure 1.**
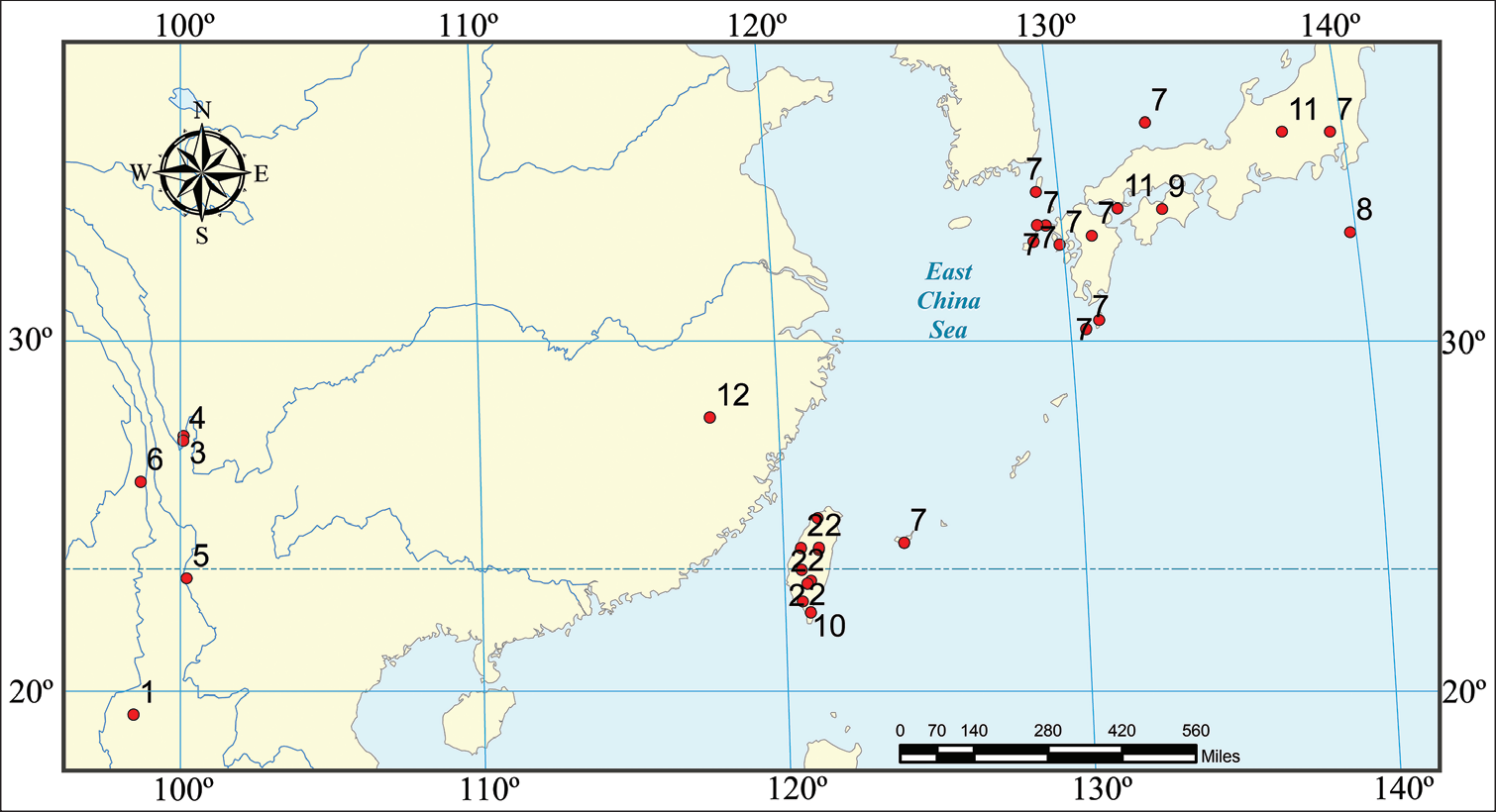
Distribution of brachypterous species of the genus *Strongylium* Kirby from China and neighbouring countries: **1** *Strongylium becvarianum* Masumoto **2** *Strongylium claudum* (Gebien) **3** *Strongylium habashanense habashanense* Masumoto **4** *Strongylium habashanense lijiangense* Masumoto **5** *Strongylium jizushanense* Masumoto **6** *Strongylium liangi* sp. n. **7** *Strongylium marseuli marseuli* Lewis **8** *Strongylium marseuli watanabei* Nomura & Yamazaki **9** *Strongylium marseuli nigripes* Nomura & Yamazaki **10** *Strongylium masatakai* Masumoto, Lee & Akita **11** *Strongylium tanakai* Ando **12** *Strongylium wuyishanense* Yuan & Ren.

Specimens were examined and illustrated under a Nikon (SMZ800) dissecting microscope (equipped with a camera lucida), illustrations were processed using the software (CorelDRAW X3). Measurements were taken using a Leica (M205 A) dissecting microscope. Habitus photographs were taken with a Nikon (D 300S) camera. The distribution data in Figure 1 are derived from examined specimens and literature records. The holotype of *Strongylium liangi* sp. n. is deposited in the Institute of Zoology, Chinese Academy of Sciences, Beijing, China (IZCAS). All other materials are in the Museum of Hebei University, Baoding, China (MHBU).

The following measurements are used in the text, with all measurements in millimeters: body length: length of the body from the anterior edge of the clypeus to elytral apex; body width: length of the maximal elytral width; pronotal length: length of the pronotum along the midline; pronotal width: maximum width of the pronotum; elytral length: length of the elytra from the base of the scutellum to the elytral apex along the suture.

## Taxonomy

### Strongylium liangi sp. n.

http://zoobank.org/A0C3D887-33D1-46F5-8123-CD3CA1901276

http://species-id.net/wiki/Strongylium_liangi

Figs 2 –10

#### Type specimen.

Holotype male: China, Yunnan, Lushui county, Pianma town, Yakou, 19.v.2005, Hong-Bin Liang leg. (IZCAS).

#### Diagnosis.

The new species is similar to *Strongylium tanakai* Ando, 2003, from Japan because their humeri are more developed than other brachypterous species of *Strongylium*, such as *Strongylium claudum* (Fig. 11) and *Strongylium wuyishanense*, but can be distinguished from the latter by its stouter body, the distance between the eyes being narrower than the transverse diameter of an eye, and the shape of the aedeagus, that is obliquely narrowed apically in dorsal view, slightly curved in lateral view.

#### Etymology.

Named in honor of Dr. Hong-Bin Liang, collector of the holotype.

#### Description.

**Figures 2−9.**
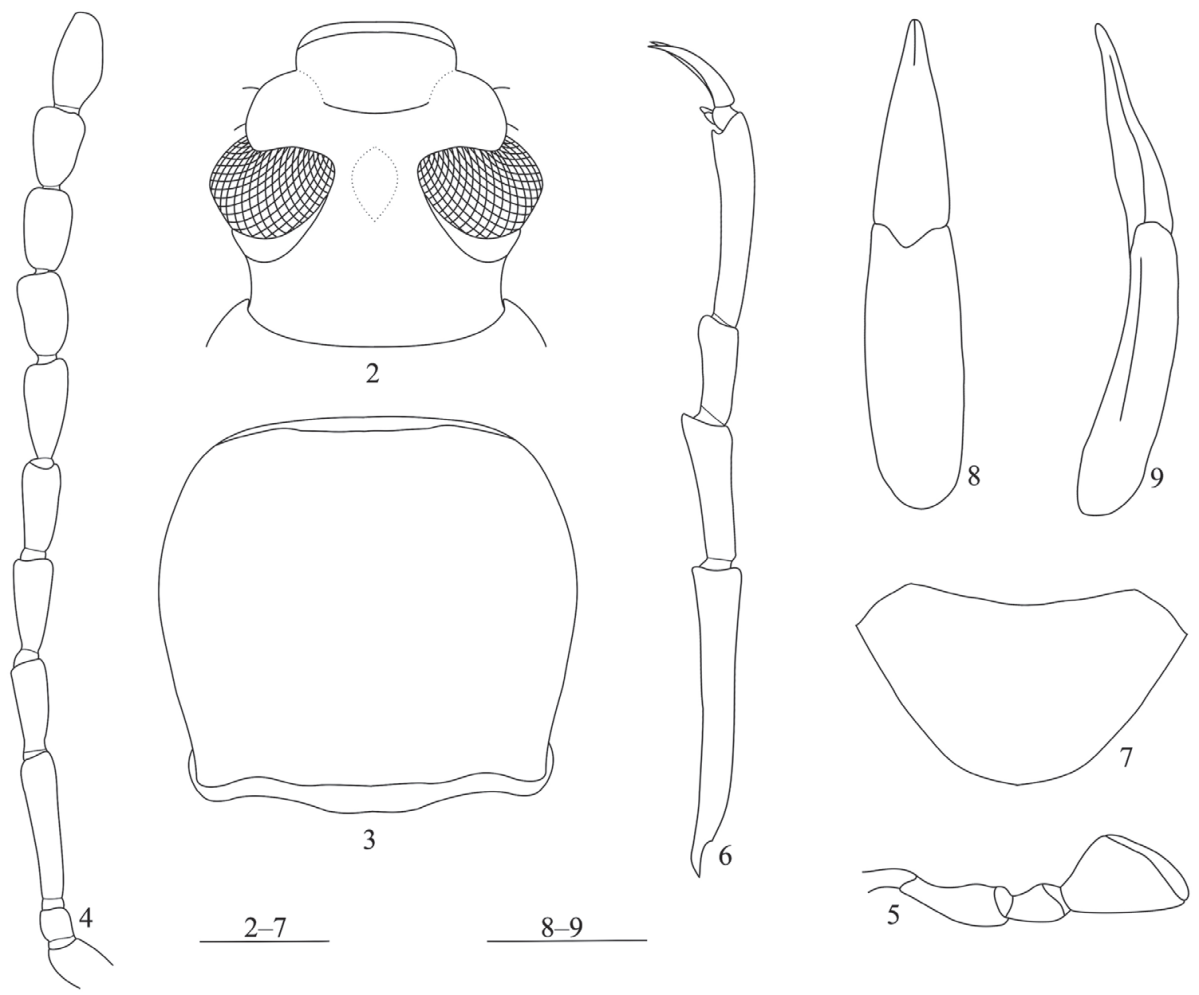
*Strongylium liangi* sp. n. **2** head **3** pronotum **4** antennae **5** maxillary palp **6** hind tibia **7** abdominal ventrite V **8** aedeagus in dorsal view **9** aedeagus in lateral view. Scales: 1 mm.

**Figures 10−11.**
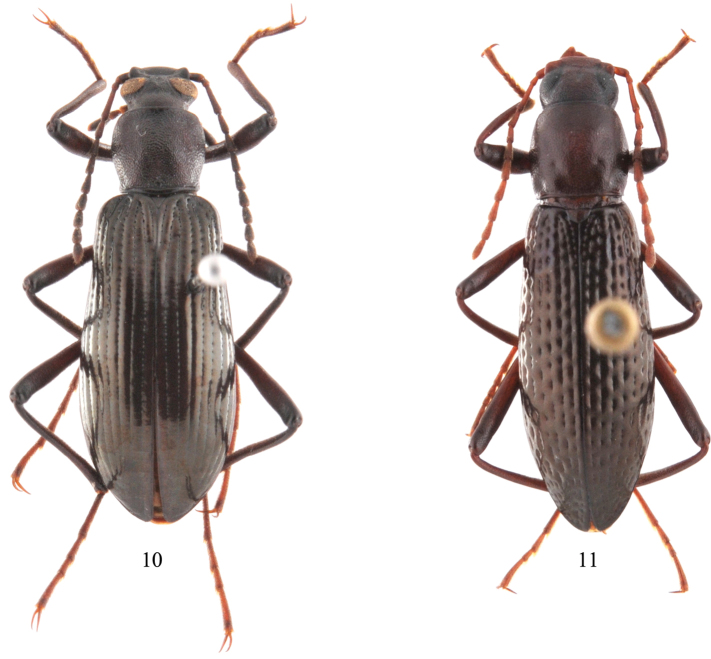
Habitus. **10** *Strongylium liangi* sp. n. **11** *Strongylium claudum* (Gebien, 1914).

**Male** (Figs 2–10). Body length 14.4 mm, elongate, slightly wider posteriorly. Colour dark brownish black, pronotum reddish, antennae and legs dark reddish brown, tarsi slightly lighter; head, antennae and pronotum almost dull, elytra shining; body surface almost glabrous except antennae, tarsi and ventral surface. Head (Fig. 2) subhexagonal, densely punctate; clypeus transverse, slightly and gradually declined forward in basal part, strongly bent ventrad in apical part, truncate at anterior edge; frontoclypeal suture deeply depressed; genae obliquely raised, with outer margins obtusely produced; frons somewhat widely T-shaped, steeply inclined anteriorly, slightly, longitudinally impressed in middle, surface irregularly and finely punctate, punctures often fused with one another, distance between eyes 0.66 times as wide as transverse diameter of an eye in dorsal view. Eyes medium-sized, rather protruding. Antennae (Fig. 4) subfiliform, reaching basal 1/5 of elytra, ratio of the length of antennomeres II–XI as 0.31 : 1.02 : 0.76 : 0.58 : 0.63 : 0.65 : 0.56 : 0.53 : 0.54 : 0.67. Maxillary palpomere IV (Fig. 5) moderately expanded. Pronotum (Fig. 3) 1.06 times as wide as long, widest before the middle; anterior margin bordered, border tapering laterad; posterior margin bisinuate, bordered; both sides steeply inclined downwards, lateral margins arcuate anteriorly, obliquely narrowed at posterior one-third, bordered along entire length; anterior angles rounded, posterior angles subrectangular; disc moderately convex, shallowly impressed near anterior margin, densely covered with confluent, ocellate punctures. Scutellum triangular, densely and rugosely punctate. Elytra elongate ovoid, slightly dilated posteriorly, 2.11 times as long as wide, widest at apical one-third, 3.68 times as long as and 1.62 times as wide as pronotum; disc slightly convex, striae fine, strial punctures circular and fine anteriorly, becoming finer and nearly disappearing apically; intervals slightly convex, flattened apically, sparsely covered with microscopic granules at posterior 1/4; humeri moderately swollen, hind wings reduced, reaching basal 3/4 of elytra. Prosternum narrow, strongly raised between coxal cavities, impressed in middle, prosternal process strongly declined to roundly produced and protruding at apex. Abdominal ventrites (Fig. 7) covered with microscopic punctures and setae, ventrite V with dense punctures and setae, setae longer than those on I–IV. Legs slender, simple, length ratio of metatarsomeres I–IV as 2.01 : 1.03 : 0.68 : 1.44. Aedeagus 2.48 mm long, 0.5 mm wide (Figs 8–9).

Female: unknown.

### Strongylium claudum

(Gebien, 1914)

http://species-id.net/wiki/Strongylium_claudum

Fig. 11

Crossoscelis clauda Gebien, 1914: 53
Strongylium claudum : Masumoto, 1999: 121.

#### Material examined.

1♂, Taiwan, Kaohsiung, Xiaoguanshan, 10.xii.1996, Wen-Yi Zhou leg.; 1♂, 1♀, Taiwan, Kaohsiung, Tengzhi, 1.xi.2008, Chang-Qing Chen leg.; 1♂, Taiwan, Pingtung, Erjituan, 5.iv.1997, Wen-Yi Zhou leg.; 1♀, Taiwan, Nantou, Ren’ai, qingjing, 1890 m, 7.v.1996, Wen-Yi Zhou leg.; 1♀, Taiwan, Taipei, Sanxia town, 24.v.1994, Wen-Yi Zhou leg.

#### Distribution.

China: Taiwan.

### Strongylium wuyishanense

Yuan & Ren, 2006

http://species-id.net/wiki/Strongylium_wuyishanense

Strongylium wuyishanense Yuan & Ren, 2006: 852.

#### Type material examined.

Holotype: 1♂ (MHBU), China, Fujian, Mt. Wuyi, Huanggangshan, 21.v.2004, Cai-Xia Yuan & Jing Li leg.

#### Distribution.

China: Fujian.

### A checklist of brachypterous species of the tribe Stenochiini from China and neighbouring countries

***Eucrossoscelis* Nakane, 1963** [Type species: *Eucrossoscelis broscosomoides* Nakane, 1963]
  (1) *Eucrossoscelis araneiformis* (Allard, 1876: 67), Japan (Nagasaki, Ryushu), (= *Strongylium helopioides* Lewis, 1894: 482) [Originally in *Helops*?; synonymized by Chûjô 1985: 65]
  (2) *Eucrossoscelis broscosomoides* Nakane, 1963: 29, Japan (Amami-Oshima Is.)
  (3) *Eucrossoscelis hastatus* Yuan & Ren, 2006: 851, China (Guizhou)
  (4) *Eucrossoscelis michioi* Chûjô, 1978: 78, Japan (Okinawa-jiama)
  (5) *Eucrossoscelis maruyamai* Masumoto, 1999: 121, Japan (Ryukyu Islands)
***Saitostrongylium* Masumoto, 1996** [Type species: *Saitostrongylium acco* Masumoto, 1996]
  (6) *Saitostrongylium acco* Masumoto, 1996: 34, Vietnam (Lai Chau)
***Stenochinus* Motschulsky, 1860** [Type species: *Stenochinus reticulatus* Motschulsky, 1860]
  (7) *Stenochinus akiyamai* Masumoto, Akita & Lee, 2013: 266, China (Taiwan)
  (8) *Stenochinus amplus* (Gebien, 1914: 8), China (Taiwan) [Originally in *Dicraeosis*]
  (9) *Stenochinus bacillus* (Marseul, 1876: 103), Japan (Nagasaki (type locality), Honshu, Shikoku, Kyushu, Okinoshima Is., Kochi Pref. and Ysushima Is.) [Originally in *Dicraeosis*]
  (10) *Stenochinus datangla* (Merkl, 1992: 273), Vietnam (Lam Dong) [Originally in *Dicraeosis*]
  (11) *Stenochinus furcifer* (Shibata, 1980: 73), China (Taiwan) [Originally in *Dicraeosis*]
  (12) *Stenochinus mysticus* Masumoto, Akita & Lee, 2013: 268, China (Taiwan)
  (13) *Stenochinus unicornis* (Shibata, 1980: 68), China (Taiwan) [Originally in *Dicraeosis*]
***Strongylium* Kirby, 1819** [Type species: *Strongylium chalconotum* Kirby, 1819]
  (14) *Strongylium becvarianum* Masumoto, 1999: 119, Thailand (Mae Hong Son)
  (15) *Strongylium claudum* (Gebien, 1914: 53), China (Taiwan) [Originally in *Crossoscelis*]
  (16) *Strongylium habashanense habashanense* Masumoto, 1999: 114, China (Yunnan)
  (17) *Strongylium habashanense lijiangense* Masumoto, 1999: 115, China (Yunnan)
  (18) *Strongylium jizushanense* Masumoto, 1999: 116, China (Yunnan)
  (19) *Strongylium liangi* sp. n., China (Yunnan)
  (20) *Strongylium marseuli marseuli* Lewis, 1894: 481, Japan (Nagasaki (type locality), SW Honshu, Oki Is., Kyushu, Tsushima, Hirado-jima, Gotô Islands, Koshiki-jima Is., Tanegashima, Ôsumi-kuroshima, Yakushima), (= *apterum* Nomura & Yamazaki, 1960: 14) [synonymized by Nakane 1975: 162]
  (21) *Strongylium marseuli nigripes* Nomura & Yamazaki, 1960: 15, Japan (Hachijô-jima of the Izu Islands)
  (22) *Strongylium marseuli watanabei* Nomura & Yamazaki, 1960: 15, Japan (Shikoku)
  (23) *Strongylium masatakai* Masumoto, Lee & Akita, 2007: 156, China (Taiwan)
  (24) *Strongylium tanakai* Ando, 2003: 79; Ando & Nakahama, 2009: 37 (male), Japan (Hyogo (type locality), Yamaguchi)
  (25) *Strongylium wuyishanense* Yuan & Ren, 2006: 852, China (Fujian)
***Uenomisolampidius* Masumoto, 1996** [Type species: *Uenomisolampidius shunichii* Masumoto, 1996]
  (26) *Uenomisolampidius shunichii* Masumoto, 1996: 36, Vietnam (Ha Tay)
***Uenostrongylium* Masumoto, 1999** [Type species: *Cryptobates laosensis* Pic, 1928]
  (27) *Uenostrongylium becvari* Masumoto, 2006: 70, China (Guizhou)
  (28) *Uenostrongylium hunanense* Masumoto, 2006: 72, China (Hunan)
  (29) *Uenostrongylium laosensis* (Pic, 1928: 26), Laos (type locality), Vietnam

## Supplementary Material

XML Treatment for Strongylium liangi

XML Treatment for Strongylium claudum

XML Treatment for Strongylium wuyishanense
